# The adulterated XANAX pill: a fatal intoxication with etizolam and caffeine

**DOI:** 10.1007/s00414-020-02352-7

**Published:** 2020-06-30

**Authors:** V. Kolbe, D. Rentsch, D. Boy, B. Schmidt, R. Kegler, A. Büttner

**Affiliations:** Institute of Legal Medicine, University Medical Center, St.-Georg-Strasse 108, 18055 Rostock, Germany

**Keywords:** Etizolam, Caffeine, Fatal intoxication, Adulterated drugs, Drug abuse

## Abstract

A 49-year old man was found dead at home next to a glass containing a dried, white, crystalline substance and near a bag containing pills with the imprint XANAX, the trade name of alprazolam. A comprehensive screening of material collected during the autopsy revealed the presence of etizolam and caffeine in lethal concentrations (0.77 μg/mL and 190 μg/mL) but no trace of alprazolam. Benzodiazepine analogue etizolam is rarely prescribed in Germany, and as a result there are not many reports about fatal cases. It has anxiolytic, hypnotic, sedative and muscle-relaxant properties and is used for the short-term treatment of anxiety and panic attacks. The purine alkaloid caffeine, conversely, is the most widely used central nervous system stimulant. The following report outlines potentially the first reported case of a lethal combination of the downer etizolam and the upper caffeine in medical literature.

## Introduction

*Etizolam* is a benzodiazepine analogue used for treating a wide range of clinical disorders. Nowadays, it is mainly used in Japan, Korea and Italy [1]. It is rarely prescribed in Germany. It has anxiolytic, anticonvulsant, hypnotic, sedative, amnesic and muscle-relaxant properties and is used for short-term treatment of insomnia, anxiety and panic attacks. Alpha-hydroxyetizolam is an active metabolite that is eliminated slowly, with a half-life of 8 h. It differs from other benzodiazepines because the molecules possess a thiophene ring instead of a benzene ring [1–3]. Cases of increased prolactin and neuroleptic malignant syndrome, which are not typical for benzodiazepines but for neuroleptics, as well as erythema annulare centrifugum have been reported [4, 5]. Etizolam is considered to be relatively safe. It has a low potential for abuse, and it is widely prescribed for the treatment of anxiety in Japan [6]. However, because of its muscular relaxing properties, it has become a serious problem in Japan in recent years due to an increase in abuse of the substance and a rise in accidents and suicides from its usage [7].

*Caffeine* is a naturally occurring purine alkaloid. It is the most widely used central nervous system stimulant in the world. It is a methylxanthine and causes the release of catecholamines, stimulating beta-1 and beta-2 adenosine receptors and inhibiting phosphodiesterase. This results in an increase in intracellular cyclic adenosine monophosphate (cAMP). It is metabolized to active dimethylxanthine stimulants theobromine and theophylline [8]. A dose of 50 to 200 mg is consistent with a mild stimulation; larger doses can result in arrhythmia, tachycardia, vomiting, convulsions, comas and death [9].

The ease in which people can access counterfeit drugs and substances is a challenging problem with severe consequences for the patient’s safety and global public health. Dangerous forms of medication are illicitly sold by transnational organizations in a broad and complex scope [10].

**Fig. 1:**
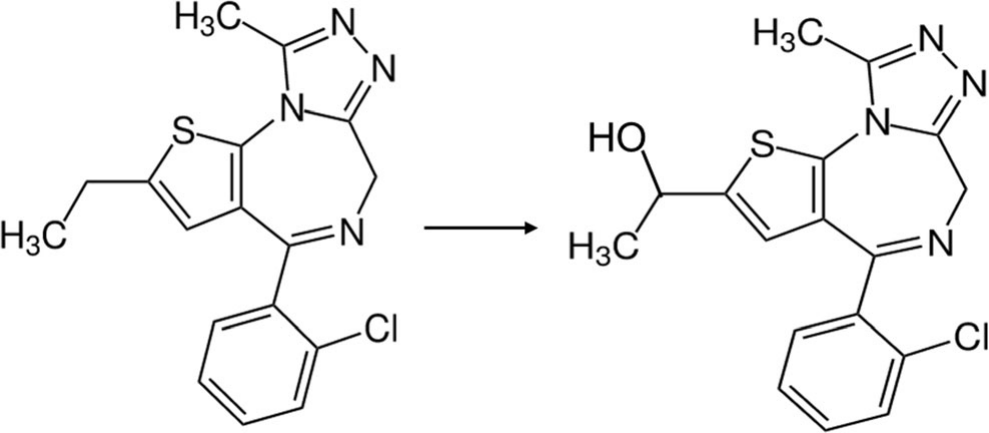
Structural formulae of etizolam and its active metabolite α-hydroxyetizolam

Fatal caffeine or etizolam intoxications are relatively rare especially because caffeine overdoses require the intake of a larger quantity. We are reporting on a case of a fatal intoxication with caffeine combined with the psychoactive substance etizolam, which we consider to be noteworthy as this combination has not been reported yet (Fig. 1).

## Case report

**Fig. 2:**
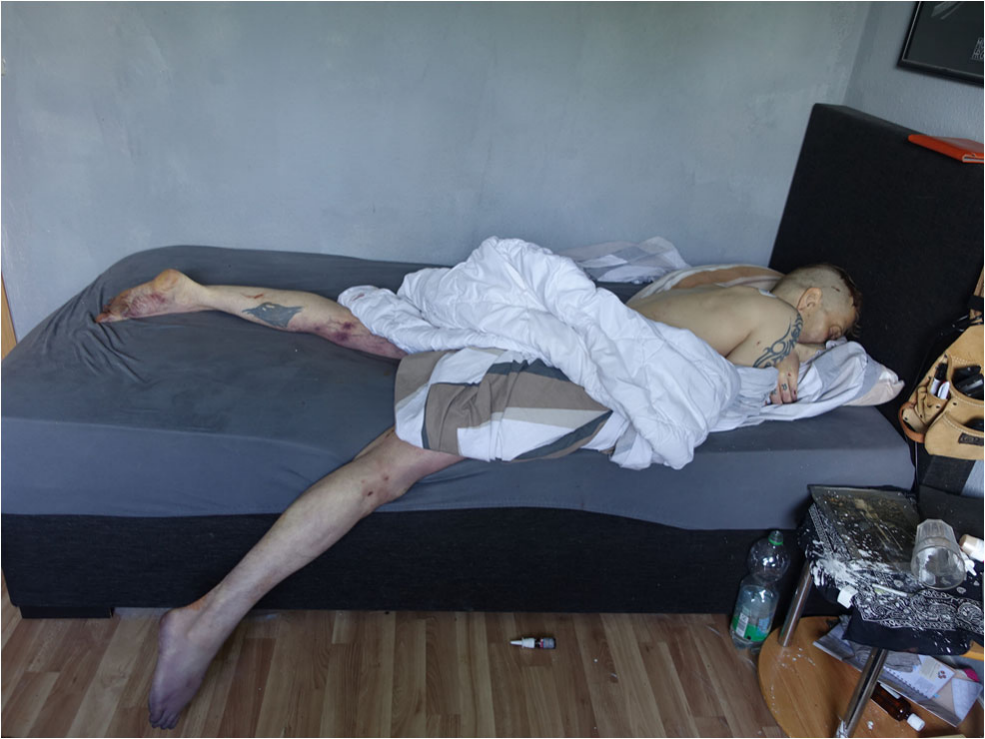
Initial situation

**Fig. 3:**
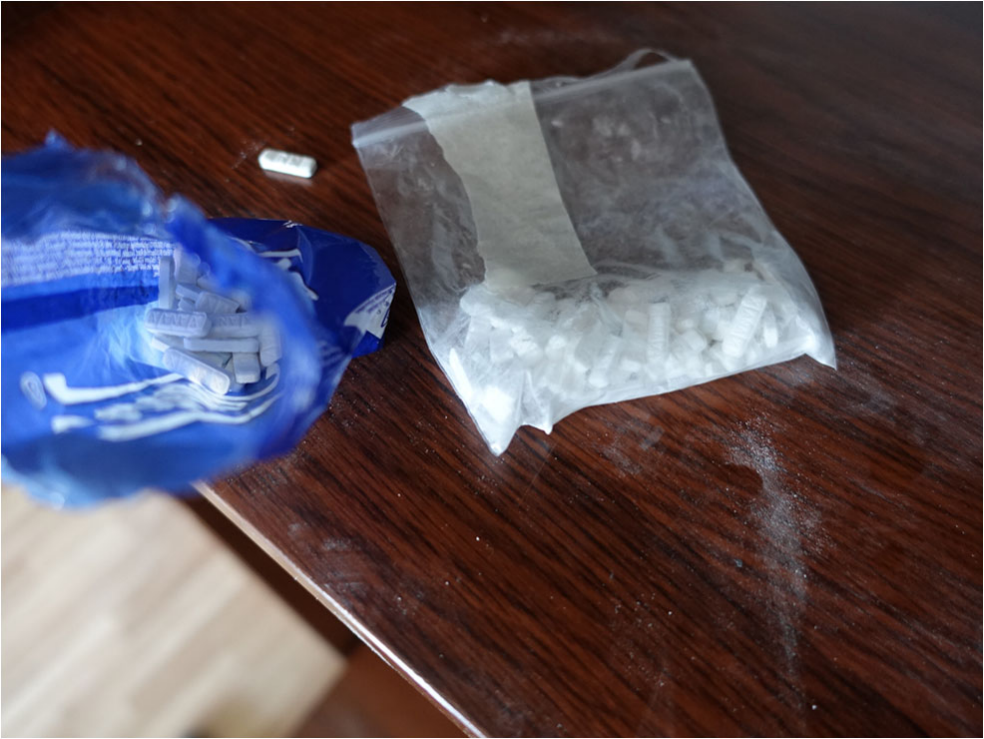
Two plastic bags containing white tablets

**Fig. 4:**
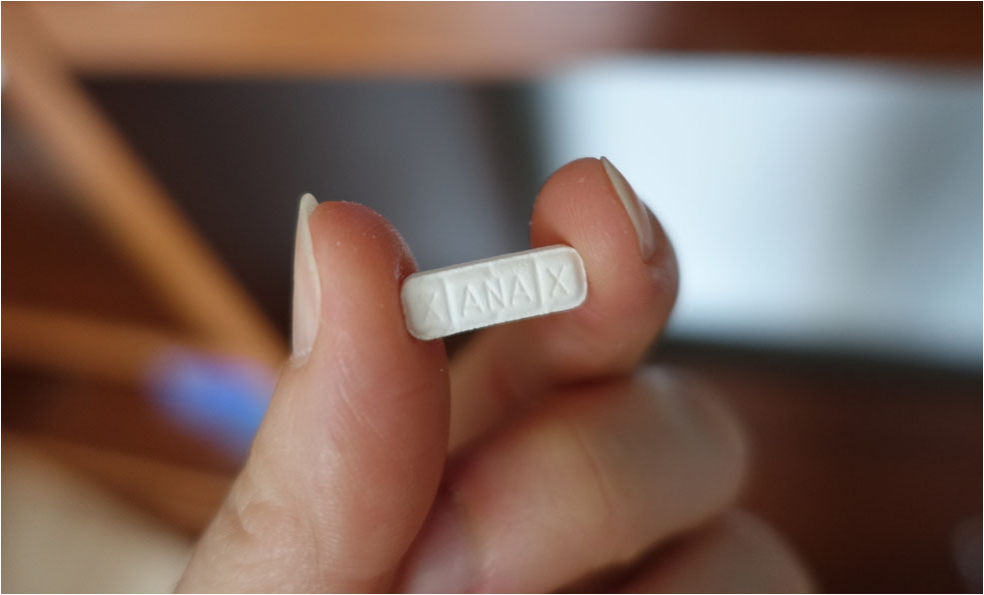
Tablet with the imprint XANAX

A 49-year-old deceased male, with a past medical history of substance abuse, was found by a friend after his separated wife could not reach him on his mobile phone. The friend, who had a key to the apartment, found the body prone on the bed in the bedroom covered in a sheet (Fig. 2). The friend notified the wife and she called an ambulance. During the first post-mortem examination, the emergency doctor noted suspected stab wounds on the man’s back and the police were notified. An examination of the deceased’s room found a toppled glass on the bedside table containing a dried, white, crystalline substance. Traces of a similar substance were found on the wall and on the floor. On a shelf in the living room, two plastic bags were found containing a large number of white tablets with the imprint XANAX (Figs. 3, and 4). In an adjoining room, there was a cannabis crop and smoking paraphernalia. The wife reported that her husband suffered from a fungal infection of the maxillary and paranasal sinus, COPD, reflux esophagitis and depression.

The forensic post-mortem examination on site showed a laceration on the scalp and some superficial scratch lesions on the back, arms, hands and legs of the man. As there was no external evidence of a penetrating trauma, the lesions on the back must have been misconstrued as stab wounds.

Besides the lacerations, the autopsy of the well-developed and nourished man revealed scars typical of self-harm, tablet-suspicious gastric content, and early signs of decay, which meant that pre-existing conditions could not be confirmed.

Histological examinations of the heart, lungs, liver and kidneys revealed signs of decomposition with the presence of gas bubbles, bacterial lawns and damaged tissue structures in the organs. The trachea was unremarkable as far as it could be observed. As no samples of vitreous humour were taken, it cannot be determined whether dehydration was a potential problem. Genetic testing for cardiac dysrhythmia can help to identify rare hereditary diseases, especially in cases with young decedents. But as the situation suggested a drug-related death, and the State authorities did not order cardiopathological diagnostics, it was not conducted. However, a postmorten DNA-analysis might have helped to identify a malignant arrhythmia.

Comprehensive toxicological tests were carried out on biological material collected during the autopsy. Body fluids were processed by solid-phase and liquid/liquid extraction. A GC-MS multi-target screening for illegal drugs and their metabolites was performed, and a general unknown screening was carried out by GC-MS and HPLC-DAD using suitable up-to-date spectral libraries (MPW5e [11], SWDRUG, Cayman, NIST, Wiley and Pragst et al. [12], respectively). Blood alcohol was determined by capillary GC-FID.

The benzodiazepine etizolam was reliably identified in the stomach content and in the powdered form via the EI-MS spectrum, the UV-spectrum and by means of a reference substance.

The quantification of etizolam in blood and urine was performed by HPLC-DAD by an external calibration after liquid/liquid extraction with n-chlorobutane in basic milieu using temazepam as internal standard. Caffeine was also quantified by HPLC-DAD after extraction with ethyl acetate under alkaline conditions using an external calibration.

In addition to etizolam, the active metabolite α-hydroxyetizolam was also qualified in urine. The quantitative examinations revealed the presence of caffeine at a concentration of 190 mg/L and etizolam at a concentration of 0.77 mg/L in femoral blood and a caffeine concentration of 426 mg/L and etizolam concentration of 2.82 mg/L in cardiac blood. THCOOH was detected at a concentration of 0.192 mg/L in the urine. The blood alcohol concentration was 0.19‰.

The approximately 5.5-cm-long dark blond hair was decontaminated with water, detergent and organic solvents and divided into four segments. The determination of common illegal drugs was carried out by GC-MS by an in-house validated method. The detection and semi-quantitative determination of etizolam in hair were performed by LC-MS after ultrasonic extraction with n-chlorobutane using etizolam-d3 as an internal standard.

Table 1 shows the concentrations of the detected substances in the investigated hair segments.

**Table 1:** Detected substances in the hair segments

Segment	Length (cm)	Etizolam (ng/mg)	THC (ng/mg)	Amphetamine (ng/mg)	Cocaine (ng/mg)	Benzoyleconine (ng/mg)
1 (incl. roots)	0.5	0.051	0.059	0.419	0.149	n.d.*
2	1.5	0.024	0.040	1318	0.034	n.d.*
3	1.5	0.037	0.130	2568	0.127	0.036
4	2	0.107	0.190	3.37	0.222	0.068

*Not detected

The results of the examined hair indicated that the descedent was an active or passive consumer of cannabis and a regular but not excessive consumer of amphetamine. The detected amount of cocain indicates a very rare consumption or an external contamination. The concentration of etizolam is difficult to interpret due to lacking reference data, but in any case it indicates multiple use. As caffeine is ubiquitous it was not determined.

Additionally, traces in the glass were analysed using the HPLC method and evidence of etizolam and caffeine were detected.

Acute toxic effects of etizolam and caffeine were determined as the cause of death.

## Discussion

Etizolam is readily available on the internet, allowing easy access to the research chemical in countries where it is not approved for general use. In patients with benzodiazepine dependence, clinicians and forensic pathologists should be aware of the potential adverse effects and the risk of reversal should always be considered [13].

Although caffeine is not usually considered as a drug because it is found in many drinks, especially caffeinated energy drinks, it has become increasingly common to consume pure caffeine as a psychoanaleptic, which creates a higher risk of severe or fatal poisoning. Single-dose caffeine fatalities have recently been reported [14]. The estimated lethal dose in an adult is 5 to 10 g, and approximately one tablespoon of pure caffeine contains about 15 g [15]. Caffeine overdoses can lead to dehydration due to severe nausea and vomiting, arterial hypertension or hypotension, muscle tremors, sinus tachycardia and cardiac arrest [8, 9, 16–19]. Intoxications may be treated with haemodialysis.

Reported fatalities involving etizolam and caffeine are summarized in Tables 2 and 3.

**Table 2:** Reported fatalities of etizolam intoxications

Sex	Age	Max. heart blood etizolam (mg/L)	Other	Authors
Female	59	0.2634	Suicide note	Nakamae et al. (2008) [6]
Male	49	0.0258	Cirrhosis	Nakamae et al. (2008) [6]

**Table 3:** Reported fatalities of caffeine intoxications

Sex	Age	Max. peripheral blood caffeine (mg/L)	Other	Authors
Female	39	192	Intravenous drug abuse	Kerrigan and Lindsey (2005) [9]
Male	29	567	Diabetes, obesity	Kerrigan and Lindsey (2005) [9]
Male	52	49	Psychiatric disorder, liver & kidney disease	Banerjee et al. (2014) [16]
Female	42	33*	Hypertension	Banerjee et al. (2014) [16]
Female	37	73*	Bulimia nervosa, diabetes, alcohol abuse	Banerjee et al. (2014) [16]
Female	39	90* (+ acetaminophen 520 mg/L and butalbital 75 mg/L)	Migraines, depression	Banerjee et al. (2014) [16]
Female	43	320*	Rheumatoid arthritis, alcohol abuse	Banerjee et al. (2014) [16]
Male	57	220	Not reported	Banerjee et al. (2014) [16]
Female	50	320	Bipolar disorder, ataxia	Banerjee et al. (2014) [16]
Male	44	74	Cardiac disease, diabetes, alcoholism	Banerjee et al. (2014) [16]
Female	19	220	Not reported	Riesselmann et al. (1999) [17]
Female	81	190 (+ salicylic acid 360 mg/L and acetaminophen 600 mg/L)	Farewell letter, pulmonary emphysema	Riesselmann et al. (1999) [17]
Female	21	not specified	Death due to pneumonia 11 days after the caffeine poisoning	Rudolph and Knudsen (2010) [19]

*Heart blood caffeine concentration as no peripheral blood was available

The present case may have resulted from a misidentification of the drug, which was declared as XANAX, the trade name of alprazolam. Alprazolam is, like etizolam, commonly used for the short-term management of anxiety disorders [20]. Reports of counterfeit Xanax tablets containing etizolam and fentanyl have been confirmed in the USA in 2016 [21]. According to Mackey et al. Asian and Latin American markets are most affected by counterfeit incidents [10].

Etizolam and caffeine can be purchased online, which makes it easy to incorporate the substances into counterfeit products. The substitution with other substances can result in unexpected reactions including death. In the case of the 49-year-old male found deceased by his friend, it remains unclear how the pills found in his residence were purchased or manufactured. The fact that the tablets were found in big plastic bags and not in trademarked packaging indicates that they may have been purchased on the internet or via other illicit means. The combination of two psychoactive substances, an upper and a downer, in ranges considered to be potentially lethal, created an unforeseeable deadly risk—the sedative effect of etizolam could have been suppressed by caffeine and masked or complicated the man’s awareness to his delirium.

The circumstances of death remained unclear: It cannot be determined whether the man died due to an intentional intoxication with suicidal intent or whether it was an accidental intake. A suicide note has not been found.

## Conclusion

This fatal case of a possibly accidental etizolam and caffeine intoxication represents a serious public health threat. There is no way for users to know exactly what they are consuming. Health care providers need to warn potential consumers about the life-threatening risks associated with taking counterfeit drugs and substances. Anonymous drug checking services that conduct forensic analysis of submitted samples could potentially save lives. This kind of surveillance strategy has already proven useful in other settings [22, 23].
